# An Update on Jacalin-Like Lectins and Their Role in Plant Defense

**DOI:** 10.3390/ijms18071592

**Published:** 2017-07-22

**Authors:** Lara Esch, Ulrich Schaffrath

**Affiliations:** Department of Plant Physiology, RWTH Aachen University, 52056 Aachen, Germany; lara.esch@rwth-aachen.de

**Keywords:** fusion protein, JRL domain, plant resistance, dirigent protein, decoy, chimeric protein

## Abstract

Plant lectins are proteins that reversibly bind carbohydrates and are assumed to play an important role in plant development and resistance. Through the binding of carbohydrate ligands, lectins are involved in the perception of environmental signals and their translation into phenotypical responses. These processes require down-stream signaling cascades, often mediated by interacting proteins. Fusing the respective genes of two interacting proteins can be a way to increase the efficiency of this process. Most recently, proteins containing jacalin-related lectin (JRL) domains became a subject of plant resistance responses research. A meta-data analysis of fusion proteins containing JRL domains across different kingdoms revealed diverse partner domains ranging from kinases to toxins. Among them, proteins containing a JRL domain and a dirigent domain occur exclusively within monocotyledonous plants and show an unexpected high range of family member expansion compared to other JRL-fusion proteins. Rice, wheat, and barley plants overexpressing OsJAC1, a member of this family, are resistant against important fungal pathogens. We discuss the possibility that JRL domains also function as a decoy in fusion proteins and help to alert plants of the presence of attacking pathogens.

## 1. Introduction

The perception of external abiotic or biotic stimuli, such as changes in temperature or microbial attack, and their translation into adaptive cellular responses is essential for all living organisms. Stimulus perception is often mediated by the binding of a ligand to a cognate receptor molecule. In plants, our understanding of receptor-ligand interactions on the molecular level has profited considerably from progress in the field of phytohormone research. Here, forward genetic screens yielding mutants with a clear developmental phenotype enabled the identification and cloning of hormone receptors in the model plant species *Arabidopsis thaliana*. A prominent example is the brassinosteroid receptor *BRI1* (*brassinosteroid insensitive 1*) that contains an extracellular leucine-rich repeat (LRR) domain (reviewed in [1]). In addition to hormone receptors, plant proteins containing LRR domains are also known as recognition proteins in plant immunity. In this context they are predicted to perceive attacking microbes and define pathogen recognition specificity [2]. In addition to LRRs, lectins are an important class of proteins involved in the recognition of microbes. Lectin proteins bind to oligosaccharides and are well known as, e.g., extracellular domains of membrane-bound receptors, and so-called lectin receptor-like kinases (LecRLKs) [3]. In this sense, LecRLKs can be seen as chimeric proteins with a binding (lectin) and an action (kinase) domain.

Most recently, our group reported on another type of chimeric, lectin-containing protein which is involved in mediating broad-spectrum disease resistance to monocotyledonous plants [4]. It was demonstrated that the lectin domain of this protein, a jacalin-like lectin (JRL), was responsible for relocating the protein towards the site of pathogen attack, most likely by binding to oligosaccharide signatures typical for the infection process. JRLs, in general, have been shown to be involved in resistance to abiotic and biotic stresses [5]. The rice protein OsJRL is up-regulated in response to salt, drought, cold, and heat stress [6]. The jacalin-related lectin RTM1 restricts the long distance movement of tobacco etch virus in *Arabidopsis thaliana* [7]. Especially, chimeric JRL proteins with a dirigent domain, like TaVER2, TaHfr-1, TaJA1, and OsJAC1 were shown to be involved in plant defense (reviewed in [8]).

In this review, we will summarize recent advances in our understanding of the role of chimeric lectin-containing proteins in plant defense, with special emphasis on those chimeric proteins containing a JRL domain.

## 2. Evolution Drives Formation of Chimeric Proteins

The majority of eukaryotic proteins consist of more than one domain, i.e., are chimeric [9]. These multiple domain proteins have diverse functions relying on the combined properties of the respective joined domains [10]. Such a gain of function by domain addition or domain swapping might lead to an evolutionary advantage. There are several mechanisms proposed about how chimeric proteins are formed. In bacteria, the fusion of genes located next to each other in the genome occurs most frequently [11]. Due to the complex intron-exon gene structure, eukaryotes employ different mechanisms for gene fusion. Typically, there is no direct joining of exons from adjacent genes involved, instead splicing patterns are modified so that a chimeric gene is transcribed from individual exons of previously independent genes [10]. Other mechanisms in eukaryotes leading to the gain of additional domains in proteins include exon extension, exon recombination, intron recombination, retroposition, and, to some extent, gene fusion [10]. The advantage of combining domains and forming chimeric proteins may be due to a potential increase in enzyme specificity or even the gain of a novel specificity, as well as the formation of physical links between single-domain proteins that cooperate to fulfill a functional role [12].

## 3. Chimeric Proteins with Lectin Domains Are Commonly Involved in Plant Defense

In general, lectins are sugar-binding proteins with at least one non-catalytic domain enabling them to selectively recognize and reversibly bind to particular carbohydrates [8]. These glycans may be present either in a free form as oligo- or polymers or as parts of glycoproteins and glycolipids. Accordingly, lectin protein families are highly diverse regarding their molecular structure and binding properties [8]. Lectins occur across all kingdoms and taxa. The most extensively studied lectins from animals are galectins [13]. They bind to β-galactoside-containing glycoconjugates and contain characteristic amino acid sequences in the carbohydrate recognition domain of the polypeptide that accounts for specific carbohydrate-binding preferences [14]. Galectins have been identified in animals but sequences encoding putative galectins have also been found in plants and viruses [15]. Another type of animal lectin is the mammalian cytosolic protein laforin which is so far the only documented phosphatase in the animal kingdom that contains an N-terminal carbohydrate-binding domain. This dual specificity enables laforins to bind complex carbohydrates such as glycogen and to remove phosphate monoesters from those sugars, thus allowing the glycogen metabolism to proceed normally and prevent the formation of insoluble Lafora bodies that are associated with the Lafora disease [16].

**Figure 1:**
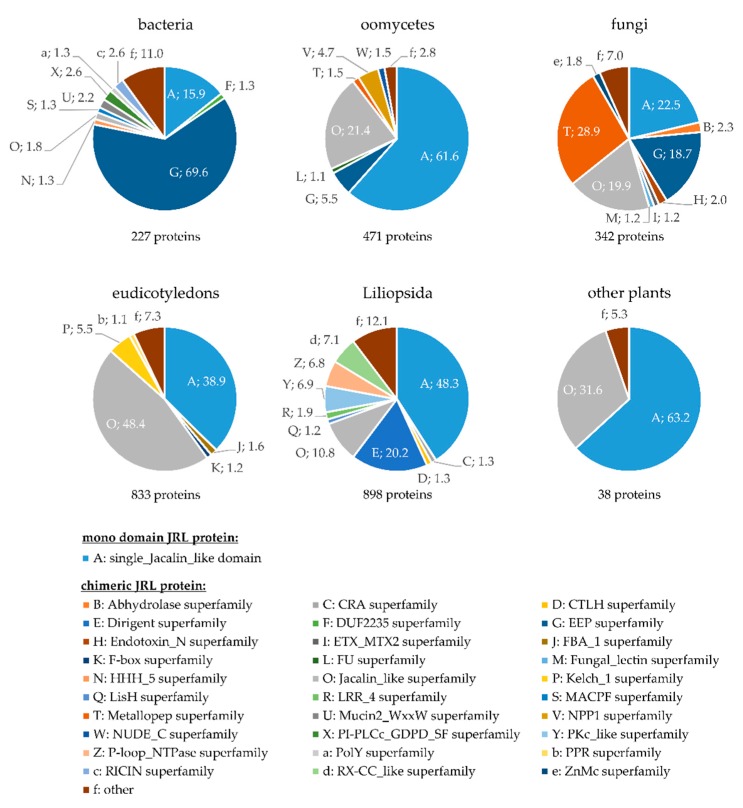
Proportional distribution of JRL domain containing proteins by superfamilies, among different kingdoms and taxa. JRL proteins (PF01419) of different taxonomic groups (bacteria, oomycetes, fungi, eudicotyledons, Liliopsida (monocotyledons) and other plants) were identified using UniProt. Domains were identified using NCBI Batch Web CD-Search Tool. Results were filtered for superfamilies as depicted. Below each pie chart the total number of JRL proteins that were found in the corresponding taxonomic group is given. Number next to the respective pie segment represent percentage of JRL domain containing proteins in depicted superfamily. Since chimeric JRL proteins may contain more than one partner domain, the respective protein might occur in different superfamilies, increasing the total percentage to >100. CRA: CT11-RanBPM; CTLH: C-terminal to LisH motif, alpha-helical motif of unknown function; DUF2235: domain of unknown function 2235; EEP: Exonuclease-Endonuclease-Phosphatase; ETX/MTX2: Clostridium epsilon toxin ETX/Bacillus mosquitocidal toxin MTX2; FBA_1: F-box associated 1; FU: Furin-like repeats; HHH_5: Helix-hairpin-helix domain; LisH: type-1-like homology motif; LRR_4: Leucin-rich repeats (2 copies); MACPF: Membrane-attack complex/Perforin domain; NPP1: necrosis-inducing protein; NUDE_C: NUDE protein, C-terminal conserved region; PI-PLCc_GDPD_SF: Catalytic domain of phosphoinositide-specific phospholipase C-like phosphodiesterases; PKc: Protein Kinases, catalytic domain; P-loop_NTPase: P-loop containing Nucleoside triphosphate hydrolases; PolY: Y-family of DNA-Polymerases; PPR: PPR repeat, unknown function; RICIN: Ricin-type beta trefoil; RX-CC: coiled-coil domain of the potato virus X resistance protein and similar proteins; ZnMc: Zinc-dependent metalloproteases.

The majority of known plant lectins consist of one or more lectin domains coupled to another, unrelated domain [8]. The most frequently occurring domain partners are annotated as dirigent, F-Box, FBA-1, kelch, PKc, RX-CC, and P-loop NTPase (Figure 1). Due to a proposed role in plant defense and development, LecRLKs, composed of an extracellular lectin, a transmembrane, and a cytosolic kinase domain, have been extensively studied during recent years. Based on the structure of the lectin domain, LecRLKs are separated in L- (legume-lectin protein-like), G- (*Galanthus nivaili* agglutinin-related), and C- (calcium-dependent) types [17]. Beside this group of membrane-bound lectins, plants produce many soluble lectins that reside in the cytosol or nucleus and are induced upon exposure to abiotic or biotic stresses. Amongst the defense-related nucleocytoplasmatic lectins, amaranthins, calreticulins/calnexins, Euonymus lectin- (EUL) related lectins, and nictaba lectins, ricin-B lectins and jacalin-related lectins (JRL) are most prominent [8].

Contrary to most lectins, the amaranthin domain itself has no sugar binding site(s); however, a specific head-to-tail arrangement of two amaranthin subunits establishes a T-antigen disaccharide binding site [18]. The nucleocytoplasmatic amaranthin of *Amaranthus* was reported to enhance resistance to aphids when ectopically expressed in transgenic tobacco and cotton by affecting the growth and development of the invading pest [19]. Calreticulins and calnexins are glucose-binding proteins, localized in the endoplasmatic reticulum (ER) of eukaryotic cells, and they act as molecular chaperones to ensure correct folding of secretory and membrane-bound glycoproteins, e.g., LecRLKs [20]. The family of EUL-related lectin genes is represented in most plants and their high degree of sequence conservation suggests an essential role for the corresponding EUL proteins [8]. Nictaba-related lectins were named after a *Nicotiana tabacum* agglutinin, originally discovered in tobacco leaves after jasmonate treatment [21]. Expression of Nictaba is enhanced in the cytoplasm upon insect attack, followed by translocation of the protein into the nucleus where it interacts with *O*-GlcNAc moieties of core histone proteins. It is hypothesized that the binding of Nictaba to chromatin results in enhanced transcription of defense-related genes [15]. The ricin B lectin family is one of the most widespread families of carbohydrate-binding proteins in nature and has been characterized in detail regarding its biological activity and toxicity in several plant species [8]. Unlike other soluble lectins, most ricin B-related proteins accumulate in the vacuole or are secreted into the apoplast [18]. Within the genus of *Sambucus* (elderberry) a variety of ricin B domain-containing proteins was identified, including SNA-I and SNA-V, and the constitutive expression of the corresponding two genes in transgenic tobacco was shown to enhance resistance to tobacco mosaic virus [22,23]. Several lines of evidence support the idea that ricin B-related lectins are also involved in plant defense against insects [24]. jacalin-related lectins (JRLs) represent a class of lectins that were first discovered in the seeds of jackfruit (*Artocarpus integrifolia* L.) [25]. JRLs are typically subdivided in galactose-specific (gJRL) and mannose-specific jacalin-related lectins (mJRL) according to their apparent monosaccharide-binding specificity [26]. Although this classification was proposed only on the basis of their monosaccharide-specificity, structural analysis of the corresponding genes supported the grouping [26].

Many JRLs have been shown to be associated with disease resistance, abiotic stress signaling, wounding, and insect damage [5]. For example, the mannose-binding rice JRL Orysata was shown to be induced upon salt stress [27]. Likewise, the recently-identified rice protein OsJRL was upregulated upon treatment with various abiotic stresses, namely salt, drought, cold, heat, and absicinic acid [6], similar to SalT [27]. In addition, the Arabidopsis JAX1 protein was found to confer resistance to a potexvirus by inhibiting the accumulation of viral RNA [28]. The jacalin-related lectin RTM1, similarly, restricts the long distance movement of tobacco etch virus in *Arabidopsis thaliana* [29]. Moreover, the mannose-specific wheat protein TaJRLL1 is considered to be a component of salicylic acid and jasmonic acid-dependent plant defense signaling mechanisms, and is activated upon infection of *Fusarium graminearum* and *Blumeria graminis* [30]. OsJAC1, particularly, plays a role in broad-spectrum resistance of rice to various pathogens [4].

Most recently, Krattinger et al. [31] identified integrated JRL domains in nucleotide-binding and leucine-rich repeat receptors ((NLR)-LRRs) of barley, rice, sorghum, and wheat. While the majority of NLRs consist of an LRR-, a transmembrane-, and a kinase domain, 10% of them contain additional domains of which some have been shown to directly interact with plant pathogens [31]. In the context of the concept of integrated decoy domains as part of plant resistance genes [32], the JRL domains of NLR-LRRs were assumed to act as decoys, trapping effectors of invading pathogens and thus enabling an adequate defense response [33]. The prominent role of JRLs in relation to biotic and abiotic stress responses, as well as their putative decoy-function, motivated us to take a closer look at this protein family.

## 4. Chimeric Proteins with Jacalin-Related Lectin (JRL) Domains Are Widely Distributed among Different Kingdoms

Proteins containing JRL domains are not exclusive to plants, but rather are present in various species of different taxonomic groups. We identified 3000 JRL domain-containing proteins in a Uniprot data base search using the Pfam domain PF01419 as query [34]. Out of these, 1756 JRL proteins were found in plants (898 in the Liliopsida, 833 in eudicotyledons, and 25 in other groups including mosses, ferns, and other spermatophytes), 471 in Oomycetes, 342 in fungi, 204 in other eukaryotes, and 227 in bacteria. Proteins consisting of a single JRL domain (single-domain JRLs) form the largest group within the eukaryota; however, chimeric JRL proteins, comprising one or more JRL domains, fused to a domain of unrelated function, are widely distributed (Figure 1). We performed a conserved domain search for the JRL proteins that were identified in the Uniprot search, using the NCBI Batch Web CD-Search Tool [35]. In this way, the domain composition of JRL domain-containing proteins is highlighted, and 172 different superfamilies that occur in combination with a JRL domain were identified. The proportional distribution of the JRL domain-containing proteins by the 172 superfamilies, among different kingdoms and taxa, was calculated and is depicted in Figure 1.

In bacteria the majority of JRL proteins (69.6%) are fusion proteins with one or more exonuclease-endonuclease-phosphatase (EEP) domain (Figure 1). The large EEP superfamily includes a diverse set of proteins that share the predicted catalytic activity of cleaving phosphodiester bonds of nucleic acids, phospholipids, and proteins [36]. EEP domain-containing JRL proteins can also be found in Oomycetes (5.2%) and fungi (18.7%) but interestingly they do not occur in plants (Figure 1). Another domain that is present in chimeric JRL proteins of fungi (28.9%) and Oomycetes (1.5%) but is not found in plants belongs to the metallopep superfamily. This protein family is widely uncharacterized but contains a metal-binding HExxH motif characteristic of metallopeptidases [37]. JRL proteins, including a necrosis-inducing *Phytophthora* protein 1 (NPP1) domain, are specific for Oomycetes and 4.7% of JRL proteins in Oomycetes comprise an NPP1 domain (Figure 1). Proteins with an NPP1 domain are known to induce necrosis in di- but not monocotyledonous plants [38]. 

In plants, single-domain JRL proteins and multi-JRL domain proteins account for the majority of proteins with a JRL domain; however, a variety of chimeric JRL proteins containing unrelated domains can be found (Figure 1). In eudicotyledons most chimeric JRL proteins contain a kelch1 domain (5.5%), an FBA_1 domain (1.6%), or an F-Box domain (1.2%), while 7.3% of the chimeric proteins are summarized in the category “other”. The kelch1 domain was first discovered as a six-fold tandem element in the sequence of the *Drosophila* kelch open reading frame (ORF) 1 protein [39,40]. The repeated kelch motif builds a conserved tertiary structure (β-propeller) that appears in different polypeptides and contains multiple potential protein-protein interaction sites [41]. The F-box domain, including the F-box associated domain (FBA_1), characterizes one of the largest protein families of eukaryotes and plays a major role in the proteolytic pathway involving ubiquitination of target proteins and their subsequent degradation by the 26S proteasome [42].

Members of the Liliopsida show the highest diversification of chimeric JRL proteins compared to plants of other classes (Figure 1). Frequently occurring domains in JRL proteins of Liliopsida are: the dirigent- (20.2%), the RX-CC like- (7.1%), the PKc_like- (6.9%), and the P-loop_NTPase domain (6.8%) (Figure 1).

Proteins containing a dirigent, Pkc, or RX-CC domain are discussed regarding their involvement in pathogen resistance. Proteins of the RX-CC domain superfamily are similar to the potato resistance protein Rx. Rx has a modular structure consisting of a coiled-coil (CC) domain, a nucleotide-binding (NB), and a leucine-rich repeat (LRR) domain, and mediates resistance to potato virus X [43]. The P-loop NTPase domain typically catalyzes the hydrolysis of the beta-gamma phosphate of bound nucleotide triphosphates (NTP) [44]. Usually P-loop NTPases show a substantial substrate preference for either ATP or GTP; however, proteins of the YchF subfamily possess the unique ability to bind both ATP and GTP. The YchF homolog from rice (OsYchF1) acts as negative regulator of plant defense responses [45].

## 5. Chimeric Dirigent-JRL Proteins Occur Exclusively in Monocotyledons (Liliopsida)

So far, we have focused on the question of which unrelated domain superfamilies occur as partners in JRL fusion proteins and how frequently they are found in different taxonomic groups. Next, we were interested in the question of whether there are specific domain compositions within the plethora of different chimeric JRL-proteins that appear noticeably more often than others, or are specific for different taxonomic groups. In Table 1 we present schematic drawings of these domain compositions encompassing the most prevalent chimeric JRL-proteins along with the total number of these proteins, as found in the previously-performed conserved domain search for the JRL domain containing proteins identified in the Uniprot query, and the taxonomic groups in which they occur.

By correlating these data, we show that while single- and multi-domain JRL proteins with up to three JRL domains are found in prokaryota and eukaryota, proteins with more than three JRL domains are only present in eukaryota (Table 1). It is predicted, because of internal sequence similarity, that the expansion of such domain repeats occur via duplication of several domains at a time [46]. Chimeric JRL proteins with one or two EEP domains followed by a single JRL domain can be found in bacteria, algae, Oomycetes, and fungi but not in plants (Table 1). Additional chimeric JRLs that are not present in plants are metallopep-JRLs, NPP1-JRLs, and ETX/MTX2 (Epsilon toxin/mosquitocidal toxin) domain-containing JRLs. ETX/MTX2 proteins are shown to be potent bacterial toxins that form channels in the cell membrane of the host leading to its death [47]. Recently, in wheat, a toxin-like pore-forming chimeric lectin, comprising two agglutinins and an ETX/MTX2 domain, was reported to confer resistance to *Fusarium* head blight [48].

Domains of plant-specific chimeric JRLs include kelch, RX-CC, ricin, PKc, or dirigent domains. Interestingly, JRL proteins with three carboxy-terminal kelch domains or an amino-terminal F-box domain are specific for eudicotyledonous plants and, to our knowledge, cannot be found in the group of monocotyledons (Liliopsida) (Table 1). In contrast, chimeric JRLs containing an N-terminal dirigent domain are specific for monocotyledons. The exclusive occurrence of specific fusion proteins within either eudicotyledons or monocotyledons indicates that the gene fusion event of the respective domains has taken place after the eudicot-monocot split which was approximately 150 million years ago [49]. The large number of dirigent-JRLs (172) suggests an essential function of these proteins in monocotyledonous plants (Table 1). During evolution, gene duplication and subsequent neo-functionalization might have contributed to the expansion of this gene family, as it has been reported that four rice dirigent-JRLs, with 62–77% sequence identity at the nucleotide level, are located directly next to each other on rice chromosome 12 [4]. Ma [50] argues that dirigent-JRLs from monocotyledonous plants have unique features compared to “classical” JRLs and proposed to name them monocot chimeric jacalins. These are exclusive to Triticeae, Oryzeae, and Andropogoneae, and evolved 50 to 25 million years ago [50].

## 6. Chimeric Dirigent-JRLs and Plant Defense

Dirigent proteins are generally assumed to play an important role in the biosynthesis of lignins and lignans [51]. In both biosynthetic pathways, dirigent proteins control the stereoselective coupling of monolignols to ensure correct formation of monolignol dimers [52]. This step is crucial, because the optical activity determines the properties of most lignans [53]. An example is gossypol, where both isomers are involved in plant defense but only the (-)-gossypol has antispermatogenic and antiviral activities, whereas the (+)-isomer is toxic to non-ruminant animals [54]. There are numerous examples that demonstrate involvement of dirigent proteins in the response of plants to pathogen attack (reviewed in [51]).

Recently, we reported our novel findings regarding the dirigent domain-containing chimeric JRL protein OsJAC1 of rice [4]. OsJAC1 is a member of the *Poaceae*-specific monocot chimeric jacalins [50]. When overexpressed in rice, OsJAC1 contributes to a broad spectrum resistance against pathogens such as *Magnaporthe oryzae*, *Rhizoctonia solanii*, or *Xanthomonas oryzae* [4]. Overexpression of OsJAC1 and the wheat/barley orthologues TaJA1 and HvJAC1 in barley similarly results in enhanced resistance to the barley powdery mildew [4]. The artificial separation of both domains and the expression of the resulting single-domain proteins diminished this phenotype, indicating the importance of both domains for full disease resistance. Localization experiments revealed that the JRL domain is responsible for relocating the OsJAC1 protein towards the infection site, possibly by binding to an infection-specific carbohydrate pattern [4].

## 7. Outlook

The Rosetta stone theory hypothesizes that fusion proteins are an indication for the cooperative activity of proteins with the respective single domains [55]. In this sense, monocot chimeric jacalins that to our knowledge have not been identified in dicotyledonous plants might point to the functional interaction of single-domain JRLs and single-domain dirigent proteins in dicotyledonous plants. Whether this is the case and if there is a link to plant immunity will be determined in future studies. Additionally, ligands that bind to monocot-specific JRLs, which are involved in plant immunity, have yet to be identified in follow-up studies. Similarly, the function of the dirigent domain of these JRLs has to be uncovered in order to reveal whether they are involved in the re-enforcement of plant cell walls or the production of anti-microbial compounds.

Both aspects—the identification of interacting pairs of dirigent and JRL proteins in dicots, as well as the characterization of the still poorly-described mode of action of monocot chimeric jacalins—will be important steps in advancing our understanding of plant resistance.

## Figures and Tables

**Table 1:** Domain composition of most prevalent single-domain and chimeric JRL proteins and their occurrence in different kingdoms and taxa. Domains were identified using NCBI Batch Web CD-Search Tool. Proteins with domain compositions occurring in more than one taxonomic group or in more than 20 proteins out of 3000 JRL domain (PF01419) containing proteins were identified with UniProt. Column one shows the protein domain composition. Columns two and three show the number of proteins (No. of proteins) with depicted domain composition and their occurrence in different taxonomic groups (Taxonomic groups).

Protein Domain Composition ^1^	No. of Proteins	Taxonomic Groups ^2^
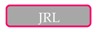	1363	B, P, A&O, F, Mn, Mv, M, Fr, oS, E, L
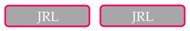	281	B, A&O, F, Mn, Mv, oS, E, L
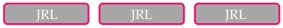	264	B, A&O, M, oS, E, L
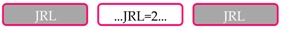	110	A&O, F, M, E, L
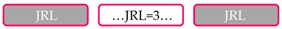	18	A&O, E
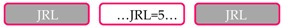	3	A&O, E
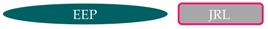	234	B, A&O, F
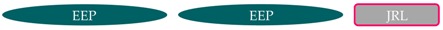	7	B, F
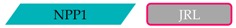	22	A&O
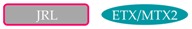	8	B, F, Mn, Mv
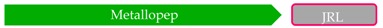	93	F, Mn
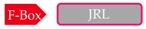	2	F, E
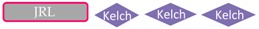	29	E
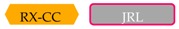	8	E, L
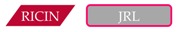	2	E, L
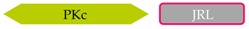	21	E, L
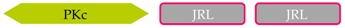	14	E, L
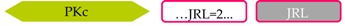	22	L
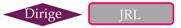	172	L

^1^ JRL: Jacalin related lectin superfamily; EEP: Exonuclease-Endonuclease-Phosphatase superfamily; ETX/MTX2: Clostridium epsilon toxin ETX/Bacillus mosquitocidal toxin MTX2 superfamily; Metallopep: Metallopep superfamily, putative peptidase family; PKc: PKc like superfamily, Protein Kinases, catalytic domain; RX-CC: RX-CC superfamily, coiled-coil domain of the potato virus X resistance protein and similar proteins; F-Box: F-Box domain superfamily; RICIN: RICIN superfamily, Ricin-type beta trefoil, Carbohydrate-binding domain; Dirigent: Dirigent superfamily, Dirigent-like protein; Kelch: Kelch-1 superfamily , Kelch motif; NPP1: NPP1 superfamily, Necrosis inducing protein; ^2^ B: Bacteria, P: Protozoon, A&O: Algae and Oomycetes, F: Fungi, Mn: Metazoa (non-vertebrata), Mv: Metazoa (vertebrata), M: Moss, Fr: Fern, oS: other Spermatophytes, E: Eudicotyledons, L: Liliopsida.

## Abbreviations

JRL	Jacalin Related Lectin
*BRI1*	*Barssinosteroid insensitive gene 1*
LRR	Leucin Rich Repeat
LecRLK	Lectin Receptor Like Kinase
FBA-1	F-Box Associated-1
PKc	Protein Kinase Catalytic Domain
RX-CC	Rx Protein-Coiled-Coil
NTPase	Nucleoside Triphosphate Hydrolase
L-type	Legum-Lectin Protein-Like Type
G-type	*Galanthus nivaili* Agglutinin-Related Type
C-type	Calcium-Dependent Type
EUL	Euonymus Lectin
ER	Endoplasmatic Reticulum
Nictaba	*Nicotiana tabacum* Agglutinin
O-GlcNAc	*o*-β-*n*-Acetyl-*d*-glucosamine
SNA-I/SNA-V	*Sambucus nigra* Agglutinin I/V
gJRL	Galactose-Specific Jacalin Related Lectin
mJRL	Mannose-Specific Jacalin Related Lectin
JAX1	Jacalin-Type Lectin Required for Potexvirus Resistance1
RNA	Ribonucleic Acid
NLR	Nod-Like Receptor
EEP	Exonuclease-Endonuclease-Phosphatase
metallopep	Metallopeptidase
NPP1	Necrosis-Inducing *Phytophthora* Protein 1
ORF	Open Reading Frame
CC	Coiled-Coil
NB	Nucleotide Binding
NTP	Nucleoside Triphosphate
ATP	Adenosine Triphosphate
GTP	Guanosine Triphosphate
ETX	Epsilon Toxin
MTX	Mosquitocidal Toxin
OsJAC1	*Oryza sativa* Jacalin-Related Lectin1
TaJA1	*Triticum aestivum* Jacalin-Related Lectin1
HcJAC1	*Hordeu vulgare* Jacalin-Related Lectin1
